# The outcome of right siting patient to primary care provider by asthma nurses

**DOI:** 10.1186/2045-7022-5-S2-P14

**Published:** 2015-03-23

**Authors:** Lai Mei Tham, Lathy Prabhakaran, Yick Hou Lim, Bing Sun, Lay Ping Neo, Sri Siti Fatimah Abdul Rashid

**Affiliations:** 1 Tan Tock Seng Hospital, Nursing, Singapore, Singapore; 2 Tan Tock Seng Hospital, Respiratory and Critical Care Medicine Department, Singapore, Singapore; 3 Tan Tock Seng Hospital, Clinical Research, Singapore, Singapore; 4 Tan Tock Seng Hospital, Office of Clinical Governance, Singapore, Singapore

## Background

Right-sitting chronic asthma care from acute hospitals to the Primary Care Provider (PCP) is an initiative undertaken at an institution in Singapore. Follow up care delivered by PCP like polyclinics (OPS) or general practitioners (GP) will aid to free hospital and Emergency Department (ED) to provide more acute and complex specialized care.

This study was to evaluate the outcome after right-sitingasthma patient from three different clinical setting (ED, In-patient and out-patient) in a tertiary institution to PCP.

## Method

It is a retrospective study from January 2012 to December 2012. The asthma nurses played pivotal role focusing on asthma education, self-management and providing support to transition patient smoothly across to PCP. Telephonic follow-up were done at 6 and 12 months. Patient’s ACT score and follow up statues with PCP were obtained. ED and hospital attendance at 6 and 12 months pre and post discharged to PCP were tracked.

## Results

A total of 460 patients were right-sited to PCP. 392 (85.2%) were decanted to polyclinics and 68 (14.8%) to GP. The mean (SD) age for patients right-sited from in-patient, out-patient and ED was 61 (19), 52 (19) and 37 (14) respectively. The attendance rate of the scheduled follow up at polyclinic and GP at 6 and 12 months were similar in both groups. The ACT scores were significantly improved for patients who were followed up at the polyclinic within 12 months after right sited to PCP (P<0.001). Out-patient had lower ED re-attendance compared to the other referral sources (p=0.006, p<0.001) and lower admission rate than in-patient (p=0.013). Re-admission patients have a statistically significantly higher mean age 55.09 than non-re-admission patients 44.38(t = -3.559, p < .001). Patient with prior ED attendance and hospitalization had higher rates of re -attendance and admission within a year (P= 0.001). The risk of ED patients to have a re-attendance at ED within 12 months was at 5 fold risk as compared to the Outpatient (p<0.001).

## Conclusions

Asthma nurse played an essential role in smooth transition of patient to the PCP. Nevertheless strategies need to be strengthened in managing ageing population and ED patient in reducing health care utilization further.

